# Supplementary material for: Serial Change of Endotoxin Tolerance in a Polymicrobial Sepsis Model
Source: Int J Mol Sci. 2022 Jun 13;23(12):6581. doi: 10.3390/ijms23126581 (PMC9223582; doi:10.3390/ijms23126581)
Supplement: Supplementary file 1 [file ijms-23-06581-s001.zip › ijms-1749856-supplementary.pdf]

Supplementary Figure S1

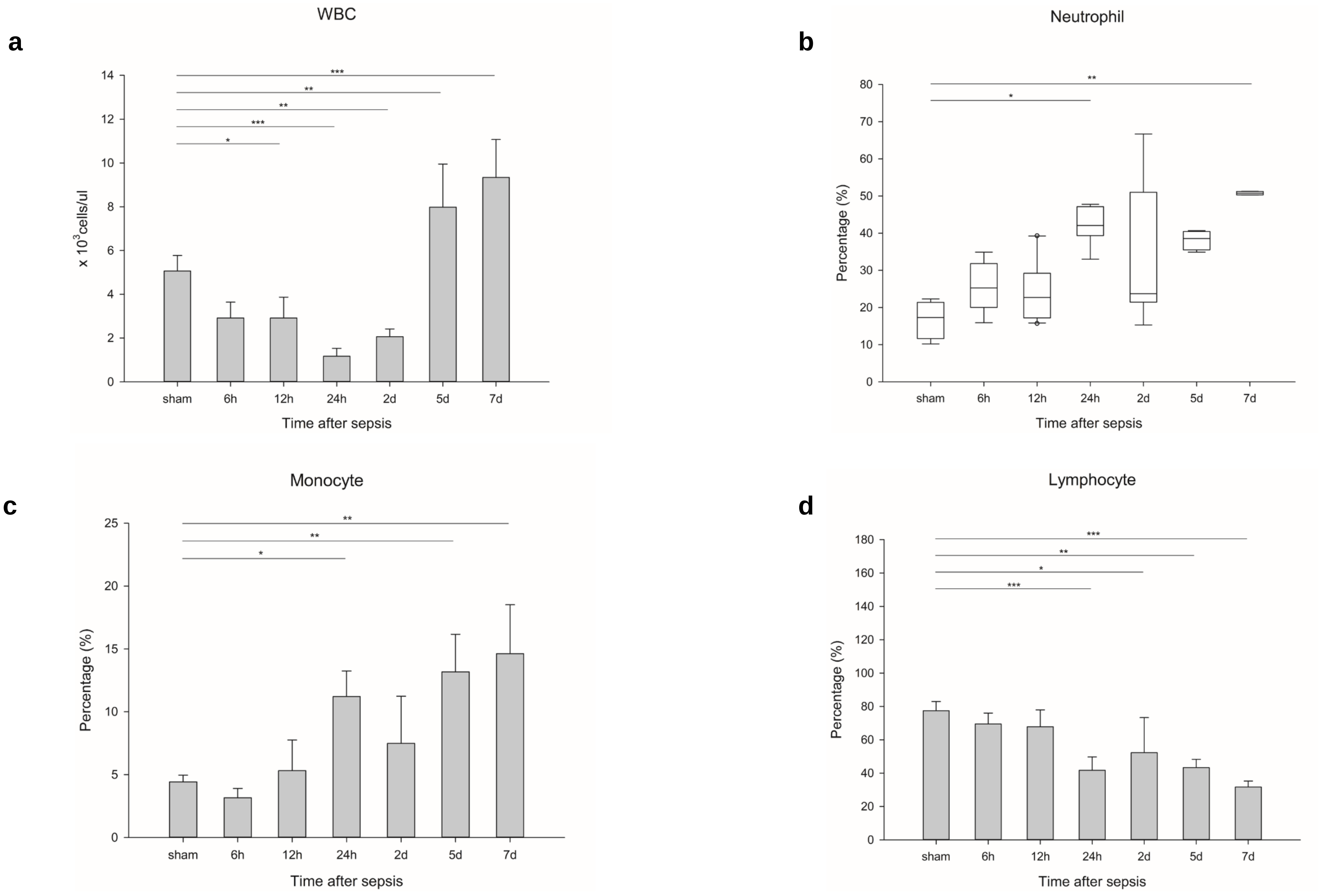

Supplementary Figure S1. Complete blood cells profiles. The type of cell profiles. Whole blood cells (WBC) (**a**), Lymphocyte (**b**), Monocyte (**c**), Neutropil (**d**). ***p <0.001 **p <0.01 and * p < 0.05 compared with the sham group.

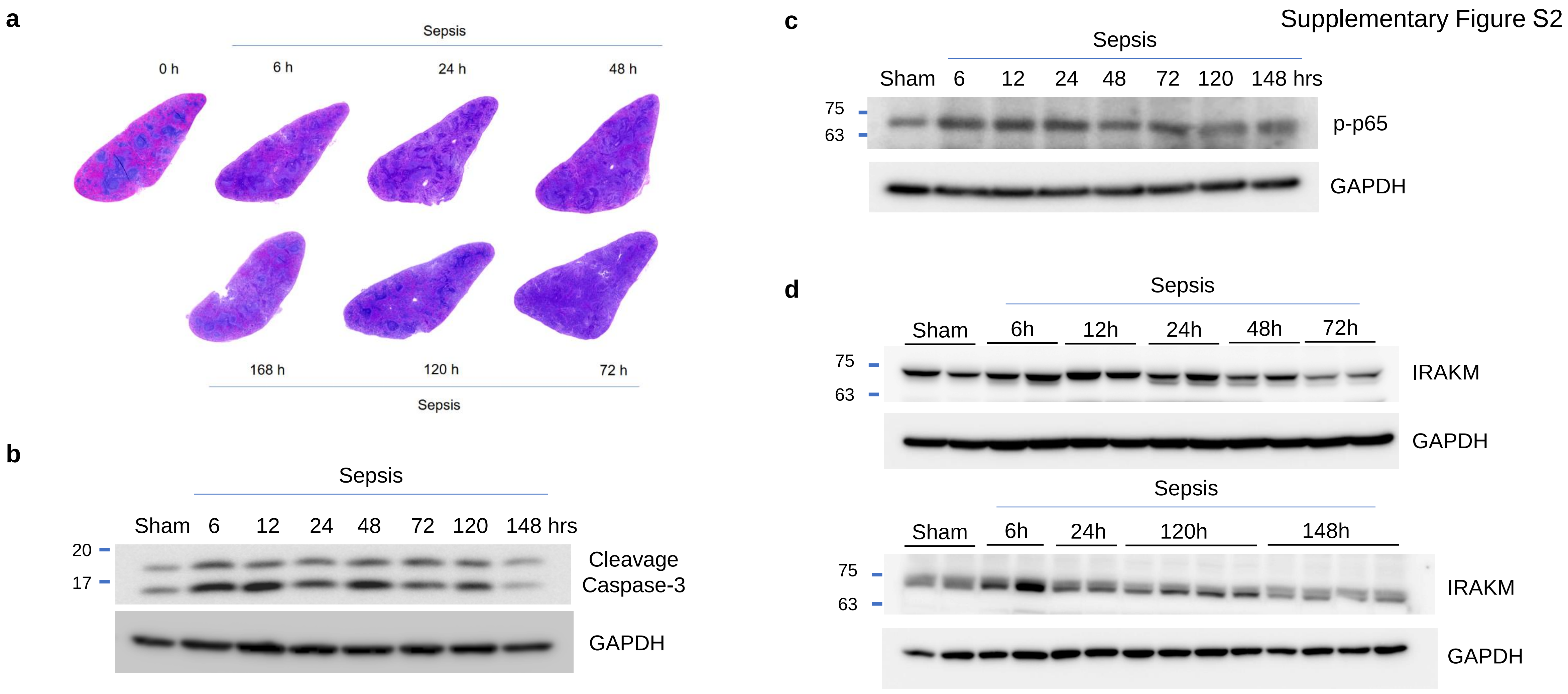

Supplementary Figure S2. Pathologic evaluation of spleen and westernblot of cleaved caspase3, p-p65, and IRAK-M. (**a**) Isolated spleens from Sham and Sepsis rats were stained with hematoxylin and eosin (H&E).The stained spleens were scanned with an AxioScan Z1(Objective:20x). Lymphoid follicle numbers were counted per 10 square millimeters of the spleen using the Zen 3.1 Blue edition program (Carl Zeiss Microscopy) in a blinded manner with three observers. (**b**) Representative images of cleaved caspase3 and GAPDH were determined with western blot (n = 3). (**c**) Representative images of phosphorylation of p65 (n = 5-6).(**d**) Representative images of IRAKM (n = 2-4). (**c**-**d**) A protein lysates for immuno-blot were extracted form spleen tissue of Sham and Sepsis rats. A detail method are described in ‘Material & Methods’.
